# A pilot study of mitochondrial genomic ancestry in admixed Brazilian patients with type 1 diabetes

**DOI:** 10.1186/s13098-024-01342-8

**Published:** 2024-06-15

**Authors:** Lívia Leite Ferreira, Anna Beatriz Rodrigues Gonçalves, Igor Josué Barbosa Adiala, Silvia Loiola, Alessandra Dias, Rossana Sousa Azulay, Dayse Aparecida Silva, Marília Brito Gomes

**Affiliations:** 1 DNA Diagnostic Laboratory, IBRAG, State University of Rio de Janeiro, Rio de Janeiro, Brazil; 2 Forensic Science and Technology Laboratory, State University of Rio de Janeiro, Rio de Janeiro, Brazil; 3 Department of Internal Medicine, Diabetes Unit, Rio de Janeiro State University (UERJ), Boulevard 28 Setembro 77, Rio de Janeiro, Rio de Janeiro Brazil; 4 Service of Endocrinology, University Hospital of the Federal University of Maranhão, São Luís, Brazil

**Keywords:** Type 1 diabetes, Mitochondrial DNA, Admixed, Population

## Abstract

Interactions between multiple genes and environmental factors could be related to the pathogenesis of type 1 diabetes (T1D). The Brazilian population results from different historical miscegenation events, resulting in a highly diverse genetic pool. This study aimed to analyze the mtDNA of patients with T1D and to investigate whether there is a relationship between maternal ancestry, self-reported color and the presence of T1D. The mtDNA control region of 204 patients with T1D residing in three geographic regions of Brazil was sequenced following the International Society for Forensic Genetics (ISFG) recommendations. We obtained a frequency of Native American matrilineal origin (43.6%), African origin (38.2%), and European origin (18.1%). For self-declared color, 42.6% of the patients with diabetes reported that they were White, 50.9% were Brown, and 5.4% were Black. Finally, when we compared the self-declaration data with maternal ancestral origin, we found that for the self-declared White group, there was a greater percentage of haplogroups of Native American origin (50.6%); for the self-declared Black group, there was a greater percentage of African haplogroups (90.9%); and for the Brown group, there was a similar percentage of Native American and African haplogroups (42.3% and 45.2%, respectively). The Brazilian population with diabetic has a maternal heritage of more than 80% Native American and African origin, corroborating the country’s colonization history.

**Supplementary Information:**

The online version contains supplementary material available at 10.1186/s13098-024-01342-8.

## Introduction

Type 1 diabetes mellitus (T1D) is an inherited polygenic chronic autoimmune disease caused by immune destruction of pancreatic beta cells, resulting in insulin deficiency [1–3]. Most genes associated with T1D susceptibility are related to the immune response [4]. Genetic variation in the region known as the human leukocyte antigen complex (HLA), especially in class II HLA antigens, is associated with genetic risk in T1D patients [4–6]. Generally, the risk conferred by HLA system genes that is more prevalent in White, Caucasian, and European-American individuals [2, 3, 5].

Another molecular marker that could be included in ancestry studies in patients with T1D is mitochondrial DNA (mtDNA). Mitochondria are membranous organelles, formed by a double membrane, present in almost all eukaryotic cells [7–9]. They are fundamental for a variety of biological functions, being responsible for regulating a wide range of cellular processes such as ATP production through oxidative phosphorylation (OXPHOS), apoptosis, β-oxidation of fatty acids, calcium homeostasis regulation, production of reactive oxygen species (ROS), and iron-sulfur cluster biogenesis [7–9].

Mitochondria have their own DNA, mtDNA, which in mammals is present in thousands of copies per cell and is inherited in a non-Mendelian maternal manner. mtDNA is a circular double-stranded DNA molecule, consisting of a heavy strand (H) and a light strand (L), without histones and organized into nucleoprotein complexes called nucleoids. It has a size of 16.5 kb, containing 37 genes responsible for encoding 13 protein subunits of the OXPHOS system, as well as two rRNAs and a set of 22 tRNAs for mitochondrial translation [7, 8]. In addition to the coding region, mtDNA contains a non-coding control region, a short displacement loop, a hypervariable segment (HVS) known as the D-loop, which houses almost all mtDNA replication and transcription [9, 10].

Thus, mtDNA has many effects, including adaptive mechanisms to deal with environmental changes and mechanisms related to cellular physiology, growth characteristics and inflammatory systems. All the abovementioned actions have a great impact on a broad range of metabolic and degenerative diseases, such as cancer, diabetes, and aging, which are related to polymorphisms and mutations in these genes [11–14].

According to Cardena, mtDNA through hypervariable regions (HVS) could be a good marker for inferring probable maternal geographic origin [15]. Brazil has important genetic diversity due to its colonization of different migratory flows and miscegenation between Native American, European, and African individuals [16, 17]. Therefore, to date, no evaluation of mtDNA in patients with T1D has been carried out in Brazil. Thus, this pilot study aimed to analyze the maternal genetic origin of patients with T1D in a highly admixed population.

## Materials and methods

**Fig. 1:**
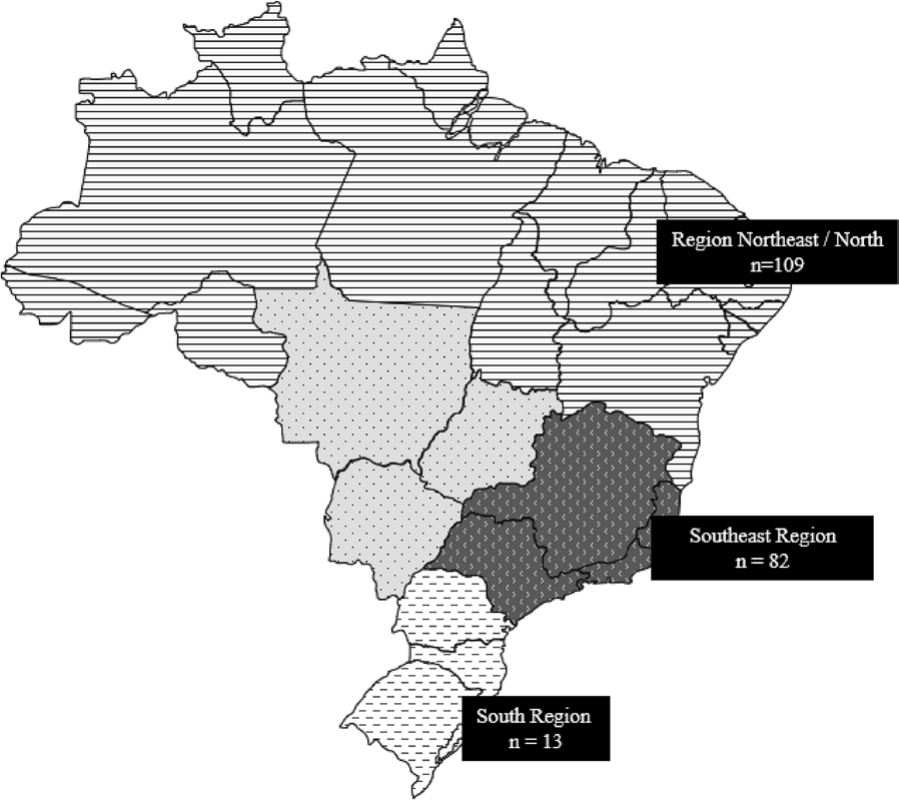
Map of Brazil's geopolitical regions with a number of samples by region: Northeast Region (102) and North Region (7), together totaling 109 samples, Southeast Region: 82 samples; South Region: 13 samples. The samples from the North Region were added to the samples from the Northeast Region, due to their low sample number

This study, with 204 anonymized and not related to each other individuals with T1D, was derived from a nationwide multicenter cross-sectional study with 1760 patients conducted in five geographical regions (North, Northeast, Midwest, Southeast and South), as previously described [1]. The present sample (*n* = 204) comprised 86 men [42.2%]; 118 women [57.8%]) who had a mean age of 27.7 (SD ± 11.28) years at diagnosis of 13.2 years (SD ± 8.29) and a mean duration of diabetes of 13.5 years (SD ± 8.43). The distribution of the studied population according to the geographic regions of Brazil is described in Fig. 1.

The control region of mtDNA, between positions 16024 to 576, was amplified and sequenced. Conventional PCR was used for amplification using the L15900 and H639 primers at a concentration of 0.2 µM each. The control region was sequenced using the L15900, L16555, L314, H159 and H639 primers at a concentration of 0.25 µM each. Electrophoresis was performed on an ABI PRISM® 3500 automatic sequencer (Applied Biosystems, CA, USA). All forward and reverse sequences were aligned with the revised Cambridge Reference Sequence (rCRS) using the SeqScape Version 2.7 program (Applied Biosystems, CA, USA) following the updated nomenclature guidelines for mtDNA. The EMPOP program was used to assign sequences to haplotypes according to the most up-to-date version of the phylogenetic tree of the mtDNA, Phylotree.

Approval was obtained before the start of the study by the Research Ethics Committee of the HUPE-UERJ (Pedro Ernesto University Hospital, State University of Rio de Janeiro) with the number CAAE 53563115.2.1001.5259. All procedures performed were in accordance with the ethical standards of the Helsinki Declaration of 1964 and later versions. Informed consent was obtained from all patients prior to recruitment.

## Results and discussion

The percentages of mtDNA haplogroups observed in patients with T1D in Brazil were Amerindian 43.6% (89 patients), African 38.2% (78 patients) and European 18.1% (37 patients). The most frequent haplogroup in the studied population was Amerindian origin C (34 patients; 16.7%), followed by African origin L3 (33 patients; 16.2%). The most common haplogroups of European ancestral origin in the population were U and J, which were observed in 8 patients (3.92%) for each haplogroup (Table 1).

**Table 1:** Haplogroup percentage in the overall studied population stratified by region

Haplogroup	N (%)	Brazilian geographic regions			Haplogroup	N (%)	Brazilian geographic regions		
		Northeast/North Region	Southeast Region	South Region			Northeast/North Region	Southeast Region	South Region
**Native American**	**89 (43.6%)**	**52 (47.7%)**	**32 (39.0%)**	**4 (30.8%)**	**Native American**	**89 (43.6%)**	**52 (47.7%)**	**32 (39.0%)**	**4 (30.8%)**
A	10.29%	8.26%	14.63%	0.00%	A	21	9	12	0
B	9.80%	11.01%	8.54%	7.69%	B	20	12	7	1
C	16.67%	19.27%	12.20%	15.38%	C	34	21	10	2
D	6.86%	9.17%	3.66%	7.69%	D	14	10	3	1
	43.63%	47.71%	39.02%	30.77%		89	52	32	4
**African**	**78 (38.2%)**	**42 (38.5%)**	**36 (44.0%)**	**1 (7.7%)**	**African**	**78 (38.2%)**	**42 (38.5%)**	**36 (44.0%)**	**1 (7.7%)**
L0	3.43%	2.75%	4.88%	0.00%	L0	7	3	4	0
L1	8.82%	6.42%	13.41%	0.00%	L1	18	7	11	0
L2	9.80%	9.17%	10.98%	7.69%	L2	20	10	9	1
L3	16.18%	20.18%	14.63%	0.00%	L3	33	22	12	0
	38.24%	38.53%	43.90%	7.69%		78	42	36	1
**European**	**37 (18.1%)**	**15 (13.8%)**	**14 (17.0%)**	**8 (61.5%)**	**European**	**37 (18.1%)**	**15 (13.8%)**	**14 (17.0%)**	**8 (61.5%)**
H	3.43%	0.92%	4.88%	15.38%	H	7	1	4	2
HV	0.98%	0.92%	1.22%	0.00%	HV	2	1	1	0
J	3.92%	3.67%	3.66%	7.69%	J	8	4	3	1
R	2.94%	3.67%	1.22%	7.69%	R	6	4	1	1
T	1.96%	0.00%	1.22%	23.08%	T	4	0	1	3
U	3.92%	4.59%	2.44%	7.69%	U	8	5	2	1
W	0.98%	0.00%	2.44%	0.00%	W	2	0	2	0
	18.14%	13.76%	17.07%	61.54%		37	15	14	8
Total	100%	100%	100%	100%	Total	204	109	82	13

Maternal lineages of African, European and Amerindian descent are expected to occur at different frequencies in Brazilian regions due to different interethnic crossings during the colonization period.

The most frequent subhaplogroup of Native American origin in the studied patients with T1D was C1b (24 patients, 11.7%), which was observed in 15 patients from the Northeast Region, 2 from the South Region and 7 from the Southeast Region. C1b is often defined as “Pan American”, as it is found in both North and South American Native Americans of Beringian origin. The C1 haplogroup was also the most frequently found in Botocudo origin (Brazil) individuals [18]. However, when comparing our results with those of other countries in South America, the most frequent haplogroup in Colombia was A2 (40%), while in our study population, it accounted for only 10.3% [18, 19].

The most frequent subhaplogroups of African origin in the studied patients with T1D were L3e and L1c (19 patients [9.3%] and 14 patients [6.8%], respectively). These subhaplogroups constitute approximately half of the African fraction of the Brazilian population (49%) studied by Alves-Silva and originated from Central Africa [20, 21].

Regarding haplogroups of European origin, our results revealed that haplogroups U and J were the most frequent (*n* = 8; 3.9% both). For the most common subhaplogroups, we found R0 (*n* = 6; 2.9%), which is frequently found in Western Europe [22].

**Fig. 2:**
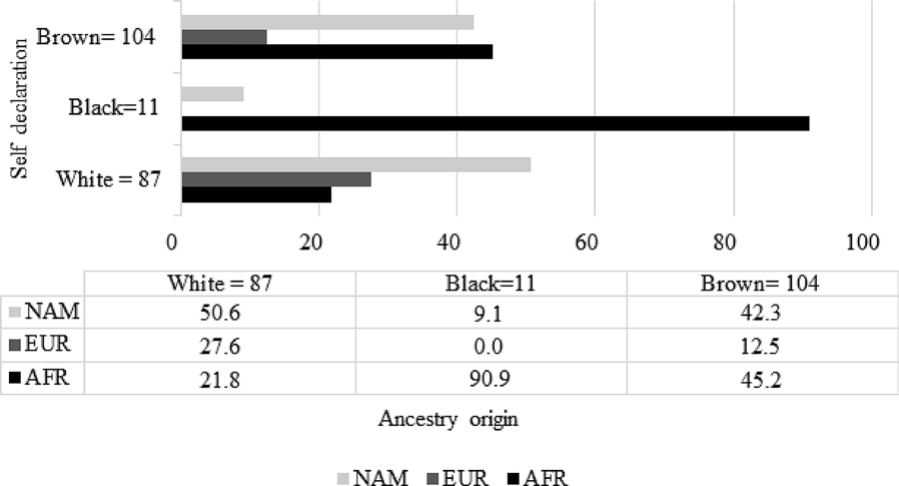
Distribution of matrilineal ancestral origin by self-reported color groups in patients with T1D (NAM: Native American; AFR: African; EUR: European)

To determine whether there is any relationship between self-reported color and matrilineal ancestry haplogroups, we determined the distribution of the ancestry origins of the matrilineal lineages (African [AFR], European [EUR] and Native America [NAM]) by groups of self-reported colors (White, Black, Brown and Yellow) in patients with T1D, as shown in Fig. 2.

In the present study, 42.6% (*n* = 87) of the patients self-declared White, 5.4% Black (*n* = 11) and 50.9% Brown (*n* = 104). Our data are similar to those of the last IBGE demographic census, 2010, which revealed that the Brazilian population comprises 47.7% Whites, 7.6% Blacks, 43% Browns, 1.1% Yellows and 0.4% Native Americans [23].

Additionally, in the present study, we observed a greater number of patients of native American origin with matrilineal ancestry. According to self-declaration, those who declared themselves White had predominantly Native American matrilineal ancestry (50.6%; 44 patients); those who declared themselves to be Brown had a similar frequency of African and Native American matrilineal ancestry (45.2% and 42.3%; 47 patients and 43 patients, respectively); and those who declared themselves Black had matrilineal ancestry of African origin (90.9%; 10 patients). ANOVA was used to validate our hypothesis, and a p value of 0.0513 indicated a tendency.

## Conclusion

The present study of patients with T1D revealed that matrilineal ancestry of Native American origin was the most common, followed by that of African and European ancestry. Our data corroborate those of other studies, confirming the influence of Brazil’s historical colonization period on the formation of the genetic pool of the Brazilian population. Further studies with a large sample of patients with T1D in all geographic regions are needed to investigate this pattern for a better definition of the role of DNAmt in the pathogenesis of the disease.

### Supplementary Information

Supplementary Material 1.

## Data Availability

The used datasets and/or analyzed during the current study are available with the corresponding author upon reasonable request.

## Abbreviations

ISFG: International Society for Forensic Genetics
T1D: Type 1 diabetes
HLA: Histocompatibility leukocyte antigen system
mtDNA: Mitocondrial DNA
HVS: Hypervariable segments
rCRS: Control region
PCR: Polymerase chain reaction
OXPHOS: Oxidative phosphorylation
AFR: African
EUR: European
NAM: Native American
