# Supplementary material for: A pilot study of mitochondrial genomic ancestry in admixed Brazilian patients with type 1 diabetes
Source: Diabetol Metab Syndr. 2024 Jun 15;16:130. doi: 10.1186/s13098-024-01342-8 (PMC11179274; doi:10.1186/s13098-024-01342-8)
Supplement: Supplementary file 1 — Supplementary Material 1. [file 13098_2024_1342_MOESM1_ESM.docx]

**Additional Table 1: Brazilian Type 1 Diabetes Study Group (BrazDiab1SG)**

Executive steering committee: Marilia Brito Gomes (chair), Carlos Antonio Negrato.

Participants and principal investigators (indicated by an asterisk).

| Marilia Brito Gomes* | State University of Rio de Janeiro | [mariliabgomes@gmail.com](mailto:mariliabgomes@gmail.com) |
| --- | --- | --- |
| Laura Nunes Melo | State University of Rio de Janeiro | [lauragnmelo@gmail.com](mailto:lauragnmelo@gmail.com) |
| Alessandra Saldanha Matheus | State University of Rio de Janeiro | [alessandramatheus79@yahoo.com](mailto:alessandramatheus79@yahoo.com) |
| Roberta Cobas | State University of Rio de Janeiro | robertacobas@gmail.com |
| Lucianne Righeti Monteiro Tannus | State University of Rio de Janeiro | luciannetannus@ig.com.br |
| Melanie Rodacki* | Federal University Hospital of Rio de Janeiro | mrodacki2001@yahoo.com.br |
| Lenita Zajdenverg | Federal University Hospital of Rio de Janeiro | lenitazaj@gmail.com |
| Joana Rodrigues Dantas | Federal University Hospital of Rio de Janeiro | joanardantasp@ig.com.br |
| Maria Lúcia Cardillo Corrêa-Giannella* | University Hospital of São Paulo | malugia@lim25fm.usp.br |
| Sharon Nina Admoni | University Hospital of São Paulo | sharonadmoni@ gmail.com |
| Daniele Pereira dos Santos | University Hospital of São Paulo | dps.daniele@ hotmail.com |
| Carlos Antonio Negrato* | Bauru’s Diabetics Association | carlosnegrato@uol.com.br |
| Maria de Fatima Guedes | Bauru’s Diabetics Association | tatiguedeses@hotmail.com |
| Sergio Atala Dib* | Federal University of São Paulo State | sergio.dib@unifesp.br |
| Celso Ferreira de Camargo Sallum Filho | Federal University of São Paulo State | celsosallum@superig.com.br |
| Paulo Henrique Morales | Federal University of São Paulo State | phmorales@institutodavisao.org.br |
| Fernando Malerbi | Federal University of São Paulo State | fernandokmalerbi@gmail.com |
| Karla Guerra Drumond | Federal University of São Paulo State | guerradrummond@gmail.com |
| Elisabeth João Pavin* | University of Campinas | [ejpavin@fcm.unicamp.br](mailto:ejpavin@fcm.unicamp.br) |
| Franz Schubert Leal | University of Campinas | franzschubertleal@gmail.com |
| Caroline Takano | University of Campinas | caroline.takano@gmail.com |
| Rosângela Roginski Rea* | Federal University of Paraná | rosangelarea@uol.com.br |
| Nicole Balster Romanzini | Federal University of Paraná | nikbr@hotmail.com |
| Mirela Azevedo* | Clinical Hospital of Porto Alegre | mirelajobimazevedo@gmail.com |
| Luis Henrique Canani | Clinical Hospital of Porto Alegre | luishenriquecanani@gmail.com |
| Felipe Mallmann | Clinical Hospital of Porto Alegre | felipekmallmann@gmail.com |
| Hermelinda Cordeiro Pedrosa* | Regional Hospital of Taguatinga | pedrosa.hc@globo.com |
| Monica Tolentino | Regional Hospital of Taguatinga | monicatolentino@uol.com.br |
| Cejana Hamu Aguiar | Regional Hospital of Taguatinga | cejanahamu@yahoo.com.br |
| André Pinheiro | Regional Hospital of Taguatinga | andrepip@gmail.com |
